# The Effect of Selective D- or N^α^-Methyl Arginine Substitution on the Activity of the Proline-Rich Antimicrobial Peptide, Chex1-Arg20

**DOI:** 10.3389/fchem.2017.00001

**Published:** 2017-01-19

**Authors:** Wenyi Li, Zhe Sun, Neil M. O'Brien-Simpson, Laszlo Otvos, Eric C. Reynolds, Mohammed A. Hossain, Frances Separovic, John D. Wade

**Affiliations:** ^1^Florey Institute of Neuroscience and Mental Health, University of MelbourneParkville, VIC, Australia; ^2^School of Chemistry, University of MelbourneParkville, VIC, Australia; ^3^Oral Health Cooperative Research Centre, Melbourne Dental School, University of MelbourneParkville, VIC, Australia; ^4^Bio21 Institute, University of MelbourneParkville, VIC, Australia; ^5^Department of Biology, Temple UniversityPhiladelphia, PA, USA

**Keywords:** A3-APO, Chex1-Arg20, D-arginine, Gram-negative bacteria, *K. pneumoniae*, backbone N^α^-methylation, proline-rich antimicrobial peptide

## Abstract

*In vivo* pharmacokinetics studies have shown that the proline-rich antimicrobial peptide, A3-APO, which is a discontinuous dimer of the peptide, Chex1-Arg20, undergoes degradation to small fragments at positions Pro6-Arg7 and Val19-Arg20. With the aim of minimizing or abolishing this degradation, a series of Chex1-Arg20 analogs were prepared *via* Fmoc/tBu solid phase peptide synthesis with D-arginine or, in some cases, peptide backbone N^α^-methylated arginine, substitution at these sites. All the peptides were tested for antibacterial activity against the Gram-negative bacterium *Klebsiella pneumoniae*. The resulting activity of position-7 substitution of Chex1-Arg20 analogs showed that arginine-7 is a crucial residue for maintaining activity against *K. pneumoniae*. However, arginine-20 substitution had a much less deleterious effect on the antibacterial activity of the peptide. Moreover, none of these peptides displayed any cytotoxicity to HEK and H-4-II-E mammalian cells. These results will aid the development of more effective and stable PrAMPs *via* judicious amino acid substitutions.

## Introduction

The increasing widespread onset of bacterial multi-drug resistance, associated with major clinical pathogenic infections, has resulted in calls for the development new antimicrobial agents (Laxminarayan et al., 2013). Due to their broad-spectrum activities and multi-modal actions against pathogens, antimicrobial peptides (AMPs) (also known host-defense peptides), are considered as attractive potential candidates for new antibiotics (Hilchie et al., 2013; Lam et al., 2016). Importantly, these peptides have also attracted considerable attention as alternative means of plant disease control to conventional treatments that are polluting and hazardous to both human health and the environment (Datta et al., 2015, 2016). Among these peptides, the class of proline-rich AMPs (PrAMPs) possess a unique multi-modal mechanism of action against pathogens and display potent activity against Gram-negative bacteria (Otvos et al., 2005; Czihal et al., 2012; Guida et al., 2015). These actions include membrane rupture (Li et al., 2014), inhibition of the bacterial shock heat protein DnaK (Kragol et al., 2001; Scocchi et al., 2009), blockade of bacterial ribosomal protein expression (Krizsan et al., 2014; Roy et al., 2015; Seefeldt et al., 2015, 2016; Goldbach et al., 2016), and immunostimulatory activity (Ostorhazi et al., 2011). Recently, a PrAMP and other AMPs were impregnated into nanofibers or hydrogels for the potential treatment of skin injuries in general and battlefield burns (Mateescu et al., 2015; Sebe et al., 2016).

The peptide, Chex1-Arg20, was *de novo* designed based on native PrAMPs with additional sequence optimization to enhance bacterial membrane penetration (Otvos et al., 2005; Noto et al., 2008; Rozgonyi et al., 2009). It has been shown that multimerization of Chex-Arg20 to a discontinuous dimer or tetramer results in an alteration of its mechanism of interaction with the *Escherichia coli* membrane (Li et al., 2015a). These observations were further confirmed on investigation of Chex1-Arg20 and its multimers with model membranes (Li et al., 2016). Additionally, specific *C*-terminal chemical modifications of the Chex1-Arg20 monomer were shown to expand both its activity and spectrum of Gram-negative bacterial action (Li et al., 2015b). These observations led to the development of a series of tetrameric Chex1-Arg20 bearing a C-terminal hydrazide that were shown to possess a more compact structure and potent and broadened activity against Gram-negative nosocomial pathogens (Li et al., 2017).

The discontinuous dimer of Chex1-Arg20, A3-APO, was shown in *in vivo* pharmacokinetic studies to undergo degradation at positions Pro6-Arg7 and Val19-Arg20, as well as to produce the major metabolite, Chex1-Arg20 (Noto et al., 2008). A key goal is to undertake chemical modifications at these labile sites to confer significant improvement in peptide stability in serum without undue effect on their activity (Otvos and Wade, 2014). D-amino acid substitution in AMPs has previously been shown to be a successful strategy (Hong et al., 1999). This suggests that partial D-amino acid substitutions within Chex1-Arg20 might be a useful means to improve its activity and stability. Furthermore, backbone N-methylation of peptide bonds can also confer high stability against proteases and improved pharmacological bioavailability (Di Gioia et al., 2016). Therefore, we undertook to incorporate the unnatural D-amino acid and N^α^-methyl-amino acid into two key points within the peptide sequence to determine the effect on activity against Gram-negative bacterium *K. pneumoniae*.

## Materials and methods

### Materials

Nine-Fluorenylmethoxylcarbonyl (Fmoc)-L-amino acids, 2-(6-chloro-1H-benzotriazole-1-yl)-1,1,3,3-tetramethylamonium hexafluorophosphate (HCTU), and 1-[Bis(dimethylamino) methylene]-1H-1,2,3-triazolo[4,5-b]pyridinium 3-oxid (HATU) were from GL Biochem (Shanghai, China). TentaGel-MB-RAM-resin was from Rapp Polymere (Tubingen, Germany). N^α^-Fmoc-N^α^-methyl-L-arginine(N^ω^-Pbf), and N^α^-Fmoc-D-arginine(D-Pbf) were purchased from Novabiochem (Sydney, Australia). N,N-Diisopropylethylamine (DIPEA), dimethylformamide (DMF), and trifluoroacetic acid (TFA) were obtained from Auspep (Melbourne, Australia). Piperidine, triisopropylsilane (TIPS), anisole, and acetonitrile (CH_3_CN) were all obtained from Sigma (Sydney, Australia).

### Peptide synthesis

The peptides were synthesized by Fmoc/tBu solid-phase methods (Fields and Noble, 1990) using a CEM Liberty microwave-assisted synthesizer and TentaGel-MB-RAM-resin as previously described (Li et al., 2015a). Standard Fmoc-chemistry was used throughout with a 4-fold molar excess of the Fmoc-protected amino acids in the presence of 4-fold HCTU and 8-fold DIPEA. For the arginine derivative substitution, 1.5-fold of amino acid coupling was used together with 1.5 equivalents HATU and 3 equivalents of DIPEA. After synthesis, the peptides were cleaved from the solid support resin with TFA in the presence of anisole and TIPS as scavengers (95:3:2, v/v) for 2 h at room temperature. After filtration to remove the resin, the filtrate was concentrated under a stream of nitrogen and the peptide products were precipitated in ice-cold diethyl ether and washed three times. The peptides were then purified by reversed-phase high performance liquid chromatography (RP-HPLC) in water and acetonitrile containing 0.1% TFA using a gradient of 10–40% (acetonitrile) in 40 min. Due to the variation in hydrophobicity between the different analogs, the final products were characterized by RP-HPLC using a gradient of either 0–40% (acetonitrile) in 40 min or 10–40% (acetonitrile) in 30 min. Matrix-assisted laser desorption/ionization time-of-flight mass spectrometry (MALDI-TOF MS) was also used for characterization.

### Antibacterial assay

An antibacterial assay was undertaken to determine the minimal inhibitory concentration (MIC) as described previously (Li et al., 2015b). The Gram-negative nosocomial bacterium, *K. pneumoniae* ATCC13883, was selected for testing the antibacterial activities of the Chex1-Arg20 analogs using 2.5 × 10^5^ cells/ml in Mueller Hinton broth (MHB) at 37°C immediately prior to the determination of MIC.

### Cell proliferation test

The proliferation of HEK-293 (ATCC® CRL-1573™) and H-4-II-E (ATCC® CRL-1548™) cells were tested with the Chex1-Arg20 analogs using the CellTiter 96 AQ_ueous_ Non-Radioactive Cell Proliferation Assay (Promega) as described previously (Li et al., 2015b).

## Results and discussion

### Peptide preparation

**Figure 1:**
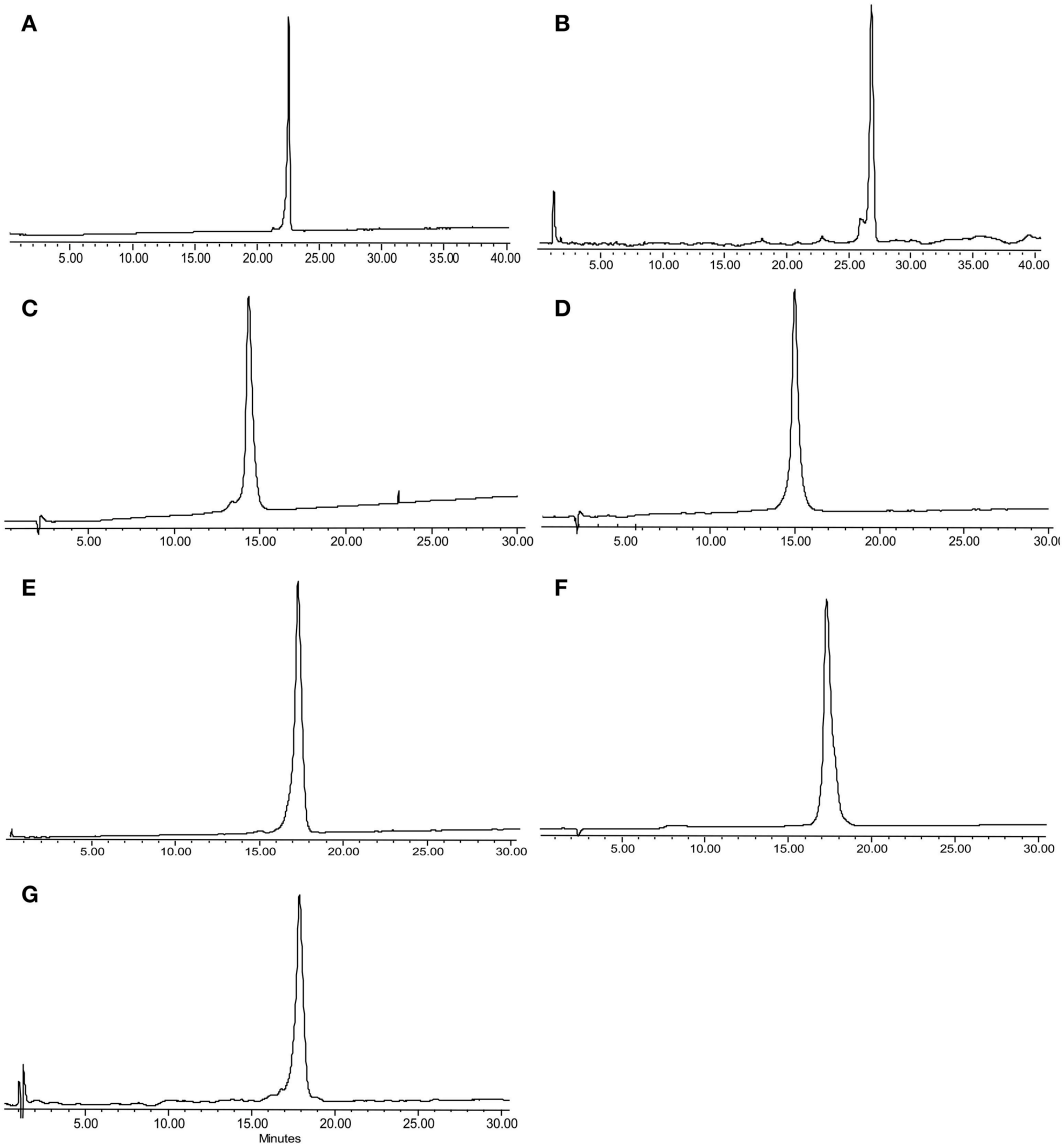
**RP-HPLC and MALDI-TOF/ESI MS for peptide analogs 2–8, respectively: (A) 2**, DR7; **(B) 3**, DR7(1–19); **(C) 4**, DR7(7–19); **(D) 5**, Chex1-Val19; **(E) 6**, DR20; **(F) 7**, mR20; **(G) 8**, reverse. Analysis condition: Phenomenex C18 column (WIDEPORE 3.6 μ XB-C18, 150 × 4.6 nm); buffer A, 0.1% aq. TFA; buffer B, 0.1% TFA in acetonitrile; gradient, buffer B 0–40% in 40 min for **(A) 2** and **(B) 3**, and 10–40% in 30 min for **(C) 4**–**(G) 8**.

Peptide **1** was prepared as described in a previous report (Li et al., 2015b) and **2–8** were prepared on TentaGel-MB-RAM-resin *via* standard Fmoc/tBu solid-phase methods. Unnatural amino acid incorporation was achieved in presence of HATU instead of HCTU (Table 1) which produced better quality products. Each Chex1-Arg20 analog was obtained in an overall yield of *ca*. ~15% relative to the crude cleaved starting material. Each analog was then subjected to comprehensive chemical characterization including analytical RP-HPLC and MALDI-TOF MS to confirm their purity (Figure 1).

**Table 1:** **Primary structure of Chex1-Arg20 analogs used in this report**.

**No**	**Name**	Sequence^*^	**MW_cal_**	**MW_fd_**
**1**	Chex1-Arg20	Chex-RPDKPRPYLPRPRPPRPVR-NH_2_	2475.0	2476.8
**2**	DR7	Chex-RPDKP r PYLPRPRPPRPVR-NH_2_	2474.9	2474.8
**3**	DR7(1–19)	RPDKP r PYLPRPRPPRPV-NH_2_	2318.8	2319.3
**4**	DR7(7–19)	r PYLPRPRPPRPV-NH_2_	1600.1	1603.0
**5**	Chex1-Val19	Chex-RPDKP r PYLPRPRPPRPV-NH_2_	2318.8	2319.2
**6**	DR20	Chex-RPDKPRPYLPRPRPPRPV r-NH_2_	2474.9	2475.2
**7**	mR20	Chex-RPDKPRPYLPRPRPPRPVmR-NH_2_	2489.0	2488.9
**8**	reverse	Chex-RVPRPPRPRPLYPRPKDPR-NH_2_	2475.0	2478.0

* *Abbreviations: r, D-Arg; mR, N^α^-methyl-arginine; MW_cal_, calculated mass; MW_fd_, found mass in MALDI*.

### Antibacterial activity

Each Chex1-Arg20 analog was assayed against the nosocomial Gram-negative bacterium *K. pneumoniae* ATCC 13883. The results are shown in Table 2 in comparison with analog **1**, Chex1-Arg20. Replacement of arginine at position 7 with the D-form (analog **2**) resulted in substantial loss of activity. This highlighted the importance of arginine-7 and its native L-configuration for characteristic antimicrobial activity. Curiously, truncation of the C-terminal Arg20 from analog 2 to produce analog **5** partially restored activity. Compared with analog **5**, the N-terminal shortened analogs **2**–**4** containing a D-arginine substitution at position 7 showed a drastic loss of activity against this pathogen in MHB. In contrast, replacement of position Arg20 with either the D-arginine or N^α^-methylated-arginine (analogs **6–7**) led to a maintenance of significant activity of the native Chex1-Arg20 which indicates that this residue is more tolerant to modification to improve its *in vivo* stability to degradation. Finally, the reverse sequence (analog **8**) was also evaluated and, as expected, it showed no activity against *K. pneumoniae* which confirmed the necessity of the native sequence for antibacterial action.

**Table 2:** **Antibacterial activity, MIC (μM), of Chex1-Arg20 analogs against Gram-negative pathogen ***K. pneumoniae*** ATCC 13883**.

**Bacterium**	1^^*^^	**2**	**3**	**4**	**5**	**6**	**7**	**8**
*K. pneumoniae*	0.8 ± 0.1^*^	>100	>100	>100	36.1 ± 0.6	11.8 ± 0.1	14.5 ± 0.1	>100

* *The activity of analog **1** was previously reported (Li et al., 2015b)*.

### Cytotoxicity

*In vitro* cytotoxicity was also measured *via* the Promega CellTiter 96 AqueousNon-Radioactive Cell Proliferation Assay (Li et al., 2015a) using the mammalian cell lines HEK-293 (ATCC CRL 1573) and H-4-II-E (ATCC CRL-1548). None of the Chex1-Arg20 analogs showed any toxicity against either mammalian cell line at the highest tested concentration (100 μM) (Table 3).

**Table 3:** **Cytoxocity (μM) of Chex1-Arg20 analogs against mammalian cell lines, H-4-II-E (ATCC® CRL-1573™) and H-4-II-E (ATCC® CRL-1548™), in which >100 μM or >50 indicated there was no cytotoxicity at the highest tested concentration 100 μM or 50 μM**.

**Analogue**	**H-4-II-E cell**	**HEK cell**
**1**	>100 μM	>100 μM
**2**	>100 μM	>100 μM
**3**	>100 μM	>100 μM
**4**	>100 μM	>100 μM
**5**	>100 μM	>100 μM
**6**	>100 μM	>100 μM
**7**	>100 μM	>100 μM
**8**	>100 μM	>100 μM

## Conclusions

In summary, a series of D-amino acid substituted analogs of the PrAMP, Chex1-Arg20, were prepared by standard Fmoc/tBu solid phase peptide synthesis. These analogs were tested against the Gram-negative bacterium *K. pneumoniae* for antibacterial activity. In this study, the activity of D-arginine Chex1-Arg20 showed the replacement of arginine at position seven led to drastic loss of activity. The short fragments, Arg2-Val19 and Arg7-Val19, also displayed no antibacterial activity. However, substitution at position 20 with either D-arginine or N^α^-methyl-arginine did not greatly affect the activity against *K. pneumoniae*. Moreover, none of these peptides showed any cytotoxicity to HEK and H-4-II-E mammalian cells. Such findings will assist the development of more effective and stable Chex1-Arg20 and A3-APO analogs with further substitution at position 20.

## Author contributions

WL performed chemical syntheses, antibacterial assay and drafted the manuscript; ZS performed cytotoxicity test; NO, LO, ER, MH, FS, and JW took part in experimental design. All authors worked on the manuscript.

### Conflict of interest statement

The authors declare that the research was conducted in the absence of any commercial or financial relationships that could be construed as a potential conflict of interest.
